# Small GTPases and phosphoinositides in the regulatory mechanisms of macropinosome formation and maturation

**DOI:** 10.3389/fphys.2014.00374

**Published:** 2014-09-30

**Authors:** Youhei Egami, Tomohiko Taguchi, Masashi Maekawa, Hiroyuki Arai, Nobukazu Araki

**Affiliations:** ^1^Department of Histology and Cell Biology, School of Medicine, Kagawa UniversityMiki, Japan; ^2^Department of Health Chemistry, Graduate School of Pharmaceutical Sciences, University of TokyoTokyo, Japan; ^3^Pathological Cell Biology Laboratory, Graduate School of Pharmaceutical Sciences, University of TokyoTokyo, Japan; ^4^Keenan Research Centre, Li Ka Shing Knowledge Institute, St. Michael's HospitalToronto, ON, Canada

**Keywords:** macropinocytosis, small GTPases, phosphoinositides, Rac1, optogenetics, myotubularin-related proteins

## Abstract

Macropinosome formation requires the sequential activation of numerous signaling pathways that coordinate the actin-driven formation of plasma membrane protrusions (ruffles) and circular ruffles (macropinocytic cups), followed by the closure of these macropinocytic cups into macropinosomes. In the process of macropinosome formation, localized productions of phosphoinositides such as PI(4,5)P_2_ and PI(3,4,5)P_3_ spatiotemporally orchestrate actin polymerization and rearrangement through recruiting and activating a variety of actin-associated proteins. In addition, the sequential activation of small GTPases, which are known to be master regulators of the actin cytoskeleton, plays a pivotal role in parallel with phosphoinositides. To complete macropinosome formation, phosphoinositide breakdown and Rho GTPase deactivation must occur in appropriate timings. After the nascent macropinosomes are formed, phosphoinositides and several Rab GTPases control macropinosome maturation by regulating vesicle trafficking and membrane fusion. In this review, we summarize recent advances in our understanding of the critical functions of phosphoinositide metabolism and small GTPases in association with their downstream effectors in macropinocytosis.

## Introduction

Macropinocytosis, an actin-dependent endocytic pathway, leads to the nonselective ingestion of extracellular fluid containing nutrients, antigens, and other molecules into cells. The process was first reported as pinocytosis (drinking by cells) by Warren H. Lewis using the earliest live-cell imaging technique, filmed time-lapse cinephotography (Lewis, 1931). Macropinocytosis, which forms large (>0.2–5 μm) endocytic vacuoles called macropinosomes, was later distinguished from micropinocytosis, which is mediated by smaller vesicles (~100 nm) such as clathrin-coated vesicles (Swanson and Watts, 1995). Compared with the mechanisms of receptor-mediated endocytosis through clathrin-coated vesicles, those of macropinocytosis have been studied less intensively in previous years. However, macropinocytosis has recently attracted increasing attention, as the important physiological and pathological implications of this process have been successively uncovered in a broad range of cell types. In macrophages and dendritic cells, exogenous proteins that are internalized through macropinocytosis are processed and presented as antigen peptides on the class II major histocompatibility complex (MHC) (Sallusto et al., 1995). In connection with pathogenesis, macrophage foam cell formation in atherosclerotic plaques can occur through the macropinocytic uptake of native low-density lipoproteins (Kruth et al., 2005). The macropinocytosis of proteins in cancer cells is a pivotal amino acid supply route for tumor growth (Commisso et al., 2013). In neuronal cells, macropinocytosis-mediated massive retrieval of the plasma membrane is an important mechanism of growth cone collapse and axon growth inhibition (Kabayama et al., 2009, 2011). Paradoxically, numerous infectious pathogens, such as bacteria, viruses, protozoa, and prions, utilize macropinocytosis for their internalization into host cells (Mercer and Helenius, 2009, 2012; Lim and Gleeson, 2011). *Salmonella typhimurium* induces membrane ruffling and macropinocytosis, which ultimately directs the internalization of the species into spacious phagosomes in host cells (Garcia-del Portillo and Finlay, 1994; Alpuche-Aranda et al., 1995). These organelles can be considered as similar to macropinosomes in the context of their formation mechanism. A better understanding of the molecular mechanisms underlying macropinocytosis, which serves several distinct purposes in different cell types, would provide new insights into a wide range of pathologies and human health issues.

The process of macropinocytosis begins with actin-driven plasma membrane ruffling. Although many ruffles recede soon after formation, some sheet-like ruffles turn into cup-shaped circular ruffles (macropinocytic cups). Then, closure of the cup opening results in the formation of intracellular macropinosomes. Subsequently, newly formed macropinosomes undergo a process of maturation for degradation or recycling. The two key events of macropinocytosis, macropinosome formation and maturation, are mediated by the actin cytoskeleton and membrane trafficking, which are primarily controlled by small GTPases and phosphoinositides (Araki et al., 2000; Cardelli, 2001; Swanson, 2008; Kerr and Teasdale, 2009; Lim and Gleeson, 2011). In this review, we summarize the regulatory relevance of small GTPase and phosphoinositide metabolism to macropinocytosis and introduce our recent findings on the implications of myotubularin-related proteins (MTMRs) in phosphoinositide metabolism during macropinosome formation.

## Small GTPases

Small GTPases of the Ras superfamily consist of several subfamilies, including the Ras, Rho, ADP ribosylation factor (Arf), and Rab GTPases, all of which are key regulators in the signaling pathways that control diverse cellular and developmental events such as differentiation, cell division, vesicle transport, nuclear assembly, and cytoskeletal organization. These GTPases are known to serve as molecular switches and regulate the process of macropinocytosis. In this section, we describe the current knowledge of the regulatory implications of each small GTPase family in macropinocytosis.

### Rho GTPases

Rho family GTPases, which act as molecular switches, regulate actin cytoskeleton remodeling through cycling between an active, GTP-bound form and inactive, GDP-bound form (Ridley, 2006). Changes in the actin cytoskeleton drive many dynamic aspects of cell behavior, including morphogenesis, migration, cytokinesis, phagocytosis, and macropinocytosis. Rho GTPases encompass three groups of proteins, Rho, Rac, and Cdc42, which differentially regulate actin-based cell structures. Typically, Rho is involved in the formation of focal adhesion and stress fibers, Rac forms lamellipodia and/or membrane ruffles, and Cdc42 forms filopodia (Nobes and Hall, 1995; Hall, 1998; Hall and Nobes, 2000). In the process of macropinocytosis, Rac1 is crucial for membrane ruffling and macropinosome formation in a variety of different cell types, including dendrite cells, macrophages, fibroblasts, and epithelial cells. Furthermore, we have recently revealed that the deactivation of Rac1 following its transient activation is also required for macropinosome formation (Fujii et al., 2013). We show these data later in a separate subsection.

Cdc42 activation has also been shown in some cell types during macropinocytosis, although its necessity to macropinocytosis has not been established (Patel and Galán, 2006). In bone marrow–derived mouse dendritic cells, the microinjection of GDP-bound inactive Rac1-T17N or treatment with *Clostridium difficile* toxin B, which inhibits all Rho GTPases (including Rac and Cdc42), abrogates membrane ruffling and macropinocytosis, whereas inactive Cdc42 does not affect macropinocytosis (West et al., 2000). GTP-bound Rac1 and Cdc42 activate p21-activated kinase 1 (PAK1), which is essential for ruffling and macropinosome formation (Dharmawardhane et al., 2000). PAK1 regulates actin cytoskeleton organization through WASP/WAVE-Arp2/3 activation and also phosphorylates the C-terminal-binding protein-1/Brefeldin A-ADP–ribosylated substrate (CtBP1/BARS), which is essential for the fission of macropinosomes from the plasma membrane (Liberali et al., 2008).

Although the precise roles of Rho isoforms in macropinocytosis remain unclear, the implications of some Rho isoforms in the process have been reported. Higher vertebrates possess three major Rho isoforms, RhoA, RhoB, and RhoC, which share 85% amino acid sequence identity. RhoA and RhoC GTPases share 92% amino acid sequence identity; however, they show different activity dynamics during macropinocytosis. Using FRET microscopy with a RhoC biosensor, RhoC was shown to be temporally activated at the circular ruffles prior to macropinosome closure (Zawistowski et al., 2013), while a burst of RhoA activity was observed after macropinosome closure in fibroblasts (Pertz et al., 2006; Zawistowski et al., 2013). Unlike RhoA and RhoC, RhoB localizes to intracellular endosomes and controls vesicle transport through regulating actin assembly on vesicle membranes (Fernandez-Borja et al., 2005); however, the specific contribution of RhoB to macropinosomes has not yet been determined. Additionally, RNAi knockdown and overexpression experiments have suggested that RhoG, a close homolog of Rac1, is required for the formation of dorsal membrane ruffles during growth factor-induced macropinocytosis in fibroblasts and A431 cells (Ellerbroek et al., 2004; Samson et al., 2010).

### Ras GTPases

The involvement of Ras in membrane ruffling and macropinocytosis has been reported in several cell types (Bar-Sagi and Feramisco, 1986; Porat-Shliom et al., 2008; Welliver and Swanson, 2012). H-Ras localizes to membrane ruffles and forms macropinosomes in epidermal growth factor (EGF)-stimulated HeLa and COS-7 cells, and the G12V active mutant of H-Ras induces membrane ruffling and macropinocytosis (Porat-Shliom et al., 2008). In RAW macrophages, Ras activation, as measured by ratio imaging of the citrine-Ras-binding domain (RBD), is observed after circular ruffle formation and is approximately concurrent with Rab5 recruitment during macropinocytosis. The peak of Ras activity occurs slightly after cup closure (Welliver and Swanson, 2012).

### Arf GTPases

The Arf family of proteins comprises another group of the Ras superfamily of small GTPases. Arf small GTPases are known to regulate membrane trafficking events, including phagocytosis and macropinocytosis, through modulating phospholipid metabolism and the actin cytoskeleton. Arf6 and Arf1, two of the best-characterized Arf proteins, have been implicated in membrane ruffling and macropinocytosis (Radhakrishna et al., 1996, 1999; Zhang et al., 1999; Schafer et al., 2000; Grimmer et al., 2002). It has been suggested that Arf6 is required for the localization of activated Rac1 to the plasma membrane. When *Salmonella* invades host cells through inducing macropinocytosis, Arf6 triggers actin assembly through recruiting ARNO, an Arf guanine nucleotide exchange factor (GEF), which activates Arf1 to enable the WAVE regulatory complex (WRC) (Humphreys et al., 2013). PI(4,5)P_2_ production by phosphatidylinositol 4-phosphate 5-kinase α (PI4P5Kα) is stimulated through the cooperation of Arf6 and Rac1 in membrane ruffle formation (Honda et al., 1999). Additionally, biochemical analysis has shown that Arf6 activates phospholipase D1, which is required for macropinosome formation (Haga et al., 2009).

### Rab GTPases

Rab GTPases are key regulators of membrane trafficking in endocytic pathways (Somsel Rodman and Wandinger-Ness, 2000; Zerial and McBride, 2001). To date, more than 60 members of the Rab family have been identified in the human genome (Colicelli, 2004). Many of the Rab proteins that are localized on distinct intracellular vesicles have been reported to coordinate sequential steps of membrane transport (Schwartz et al., 2007). In the macropinocytic pathway, several Rab GTPases have been shown to be involved in macropinosome formation or subsequent macropinosome maturation. Live-cell imaging analysis and experiments with dominant-negative mutants and RNAi have revealed that Rab5 and Rab34 are involved in macropinosome formation. Porat-Shliom et al. showed that the recruitment of Rab5 to the plasma membrane overlaps with the production of phosphatidylinositol 3,4,5-triphosphate (PI(3,4,5)P_3_) in COS-7 cells expressing H-Ras-G12V (GTP-bound form) (Porat-Shliom et al., 2008). In MEF cells stimulated with platelet-derived growth factor (PDGF), the expression of Rab5-S34N (GDP-bound form) inhibits the induction of circular ruffles (macropinocytic cups), which are the precursors of macropinosomes (Lanzetti et al., 2004). Rab5 is recruited to macropinocytic cups together with RN-tre, a Rab5 GTPase-activating protein (GAP) and Rab5 effector, which mediates actin remodeling. Supporting the involvement of Rab5 in macropinosome formation, the expression of Rabankyrin-5, another Rab5 effector, promotes fluid-phase uptake in EGF-treated A431 cells (Schnatwinkel et al., 2004). FRET microscopy has shown that Rab5a activation follows immediately after its recruitment to nascent macropinosomes in growth factor–stimulated COS-7 cells and macrophages. Rab5a activity increases temporally in early macropinosomes and then decreases prior to its dissociation. The overexpression of activating and inhibitory proteins indicates that active Rab5a stabilizes macropinosomes (Feliciano et al., 2011).

During macropinocytosis, Rab34 is also associated with actin-rich membrane ruffles and regulates macropinosome formation. In mouse embryo fibroblast (C3H 10T1/2) cells, Rab34-mediated macropinocytosis requires the activity of Rac1 and the actin nucleation factor WAVE2 (Sun et al., 2003). However, when Coxa virus enter human intestinal epithelial Caco-2 cells through macropinocytic activity, Rab34-mediateded macropinocytosis is dependent on Ras but not Rac1 (Coyne et al., 2007).

**Figure 1:**
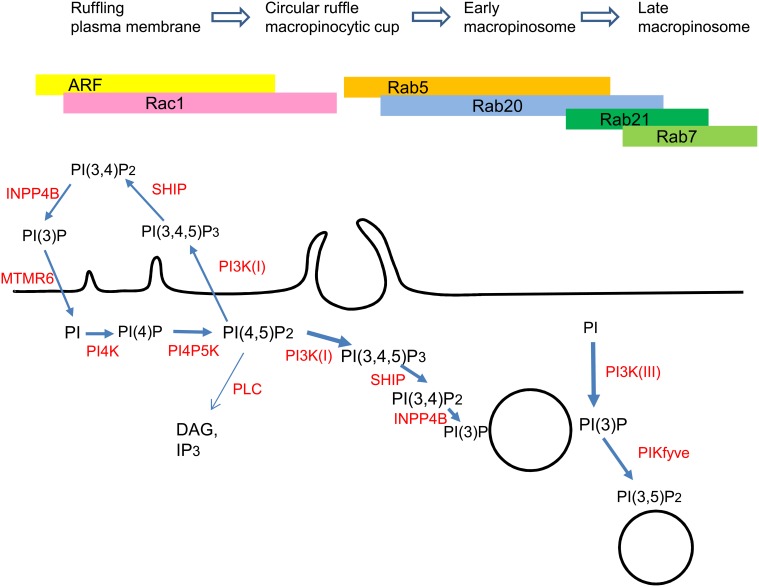
**Proposed model for the sequential association of small GTPases and phosphoinositides with the processes of macropinocytosis**. See text for explanations.

In the process of macropinosome maturation, internalized macropinosomes migrate in a centripetal manner, contracting and rapidly acquiring late endosomal/lysosomal markers such as Rab7 (Racoosin and Swanson, 1993; Kerr et al., 2006). Although the physiological roles of each Rab protein in macropinosome maturation remain undefined, several Rab proteins are associated with macropinosomes at different points during macropinosome maturation (Figure 1). Our time-lapse observations revealed that Rab21 and Rab20 are recruited to Rab7-positive maturing macropinosomes in RAW264 macrophages (Egami and Araki, 2009, 2012). Rab21 and Rab20 are close homologs of Rab5 (Schwartz et al., 2007). These GTPases show similar but not identical spatiotemporal dynamics during macropinocytosis. Although Rab21 is largely colocalized with Rab5, the recruitment of Rab21 to the macropinosomes lags a minute behind that of Rab5 and precedes that of Rab7 (Egami and Araki, 2009). The difference between Rab5 and Rab21 is further emphasized by the recruitment of Rab21 to the macropinosomes after a decrease in PI(3,4,5)P_3_ levels. Rab21 then dissociates from the macropinosomes prior to the accumulation of Lamp1, a late endosomal/lysosomal protein. Notably, the expression of Rab21-T33N (GDP-bound form) does not inhibit macropinocytic cups or macropinosome formation, suggesting that Rab21 is unnecessary for macropinosome formation. Similarly to Rab21, Rab20 is also localized to macropinosomes. Although Rab20 is colocalized with Rab5 and Rab21 at the macropinosomal membranes, the association of Rab20 with the macropinosomes persists even after the dissociation of Rab5 and Rab21 (Egami and Araki, 2012). Intriguingly, Rab20 is colocalized both with Rab7 and Lamp1 on the macropinosomes. At present, the roles of multiple additional Rab GTPases in macropinocytosis remain under investigation.

### The significant role of Rac1 deactivation during macropinosome formation

Until recently, most studies have focused on the activation (switching on) of Rho family GTPases in membrane ruffling and macropinocytosis. However, the pivotal role of the deactivation (switching off) of these molecular switches in the completion of macropinocytosis is now becoming clear. Yoshida et al. showed the spatiotemporal activation of Rac1 during macropinosome formation in live macrophages using FRET image analysis, suggesting that temporal activation followed by deactivation may be important in the process (Yoshida et al., 2009).

**Figure 2:**
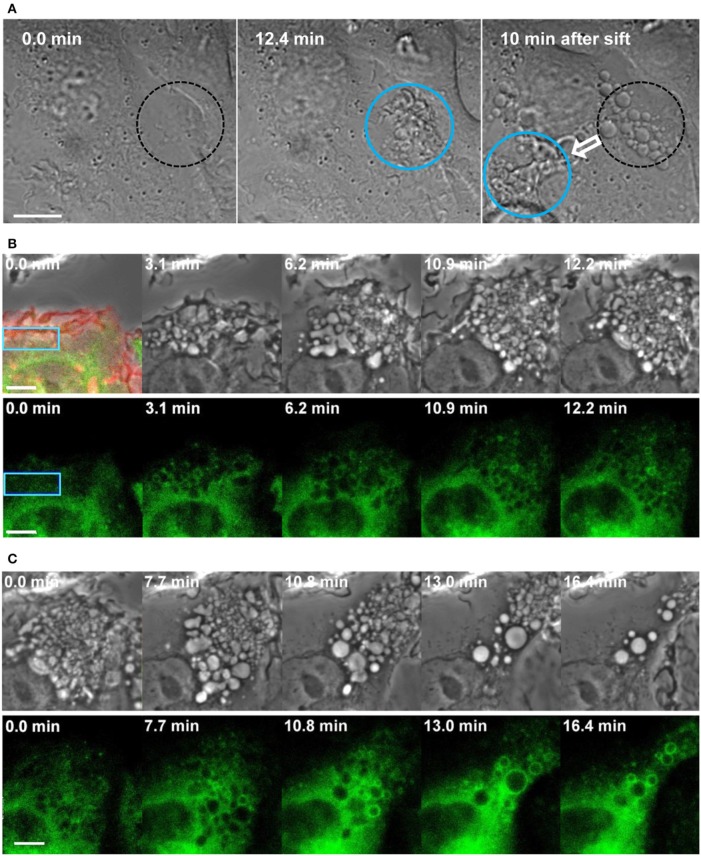
**Macropinosome formation and maturation by optogenetic control of Rac1 activation and deactivation. (A)** Local and reversible control of membrane ruffling and macropinosome formation through Rac1 photoactivation and deactivation. Diffraction interference contrast images of live RAW264 macrophages expressing PA-Rac1 were acquired by confocal laser microscopy with photoactivation. Time 0 indicates the initiation of blue laser irradiation in the area enclosed by the circle. At 12.4 min, ruffles were apparent within the irradiated region (blue circle). After 13 min, the irradiation was shifted to a different area of the same cell. After the irradiation was ceased, the ruffles immediately receded, and spherical macropinosomes were formed in the initial irradiation area (broken-lined circle) within 10 min. Marked ruffling was then induced in the newly irradiated area. The bar indicates 10 μm. **(B,C)** Time-lapse live-cell images of RAW264 macrophages expressing PA-Rac1 and GFP-Rab21 during photoactivation **(B)** and after photoactivation was ceased **(C)**. The blue rectangular area in the cell was repeatedly irradiated using a blue laser for 12.2 min. The top left panel shows a merged image of the phase-contrast, mCherry, and GFP fluorescence signals obtained before the photoactivation of PA-Rac1, confirming the expression of mCherry-PA-Rac1 and GFP-Rab21. The other panels show selected time-lapse phase-contrast (upper) and GFP-Rab21 images (lower; green). Time 0 indicates the initiation of photoactivation **(B)** or deactivation. **(C)** The recruitment of Rab21, a maturation marker, to the macropinosomes was found after PA-Rac1 deactivation. The bar indicates 5 μm (adapted from Fujii et al., 2013).

In addition, using optogenetics to manipulate Rac1 activity through blue laser irradiation, we dissected the roles of Rac1 activation and deactivation in the process of macropinocytosis (Fujii et al., 2013). Genetically encoded photoactivatable Rac1 (PA-Rac1), which is a fusion of the dominant active Rac1 with the light oxygen voltage (LOV) domain and Jα-chain (Wu et al., 2009), can be locally activated in live macrophages by blue laser irradiation under a confocal microscope. When we irradiated a peripheral portion of the cell, remarkable cell surface ruffling was induced in the irradiated area. In addition, enhanced PI(4,5)P_2_ production and actin assembly were confirmed at the region of PA-Rac1. Soon after irradiation was ceased, ruffling receded; macropinosomes were then formed from the cup or pocket-shaped ruffles (Figures 2A,B). Markers of macropinosome maturation, such as PI(3)P and Rab21, were recruited to the formed macropinosomes after the irradiation was turned off (Figure 2C). However, when we continued the irradiation, macropinosome formation was not observed; cup or pocket-like structures instead accumulated at the photoactivation region (Fujii et al., 2013; Araki et al., 2014). Thus, Rac1 activation alone is insufficient for closing macropinocytic cups into macropinosomes, although it efficiently forms circular ruffles. These results suggest that deactivation following the activation of Rac1 is crucial to the completion of macropinosome formation.

## Phosphoinositides and lipid-modifying enzymes

Phosphoinositides are produced by the phosphorylation of phosphatidylinositol (PI) on the 3, 4, and 5 positions of its inositol ring. Phosphoinositides comprise only a small fraction of cellular membrane phospholipids but are involved in many cellular processes, such as signal transduction and membrane dynamics (Di Paolo and De Camilli, 2006). Each phosphoinositide recruits specific proteins to the membrane domain where the phosphoinositide is enriched, then activates protein function and leads signaling cascades. The processes of macropinocytosis seem to be tightly regulated by a series of phosphoinositides with precise spatiotemporal patterns. In this section, we review the involvement of phosphoinositides in membrane ruffling, macropinocytic cup formation, macropinosome formation, and macropinosome maturation. Our recent observations of *Caenorhabditis elegans*, which revealed critical phosphatases that regulate phosphoinositide metabolism in membrane ruffles (Maekawa et al., 2014), are also described.

### Phosphatidylinositol 4,5-bisphosphate (PI(4,5)P_2_) and phosphatidylinositol 4-phosphate 5-kinases (PI4P5K) in membrane ruffling

PI4P5K catalyzes the phosphorylation of phosphatidylinositol 4-phosphate (PI4P) to form phosphatidylinositol 4,5-bisphosphate (PI(4,5)P_2_). Live-cell imaging using a fluorescent protein–fused PLC-PH domain demonstrated that the levels of PI(4,5)P_2_ are markedly increased in membrane ruffles and macropinocytic cups (Araki et al., 2007; Welliver and Swanson, 2012). Therefore, PI4P5K must be locally activated for localized PI(4,5)P_2_ synthesis from PI4P. In support of this finding, both Rac1 and ARF6, which activate PI4P5K activity (Honda et al., 1999), are known to be temporally activated at the same region (Zhang et al., 1999; Balañá et al., 2005; Yoshida et al., 2009). PI(4,5)P_2_ binds to activate various actin-binding proteins and initiates the actin polymerization and reorganization required for membrane ruffling, which is a prerequisite for macropinosome formation. Furthermore, PI(4,5)P_2_ is the most common physiological substrate for phospholipase C (PLC), which produces two important messengers: diacylglycerol (DAG) and inositol trisphosphate (IP_3_). DAG activates a number of protein kinase C (PKC) isoforms and causes their translocation to the plasma membrane from the cytosol. PKC activation by phorbol ester is known to enhance membrane ruffling and macropinocytosis (Swanson, 1989). The PI(4,5)P_2_ levels in the membrane decrease upon macropinosome closure. At that time, PI(4,5)P_2_ is hydrolyzed to IP_3_ and DAG by phospholipase Cγ (PLCγ) or converted to PI(3,4,5)P_3_ by PI3K during the process of macropinosome formation. However, PLCγ and PI3K seem to function in distinct phases of macropinocytosis. The inhibition of PLCγ by U-73122 perturbs macropinocytosis at the ruffle formation step, while PI3K inhibitors block macropinocytic cup closure without affecting membrane ruffling (Araki et al., 2007). The depletion of PI(4,5)P_2_ by PI3K and PLC may be important for the localized release of PI(4,5)P_2_-binding proteins from the membrane (Terebiznik et al., 2002; Scott et al., 2005).

### Phosphatidylinositol 3,4,5-trisphosphate (PI(3,4,5)P_3_) and class I phosphatidylinositol 3-kinases (PI3Ks) in macropinosome formation

The necessity of PI3K for membrane ruffling varies among different cell types and receptors. Although PDGF-induced membrane ruffling in fibroblasts is eliminated by PI3K inhibitors, membrane ruffling and circular ruffle formation in macrophages and EGF-stimulated A431 cells are resistant to these inhibitors (Araki et al., 1996, 2006, 2007). The pivotal role of class I PI3K for macropinosome closure has been well established in several cell types (Araki et al., 1996, 2006; Amyere et al., 2000).

Using live-cell imaging and scanning electron microscopy of macrophages and EGF-stimulated A431 cells, we showed that PI3K inhibitors such as LY294002 inhibit macropinosome formation but not membrane ruffling or actin assembly (Araki et al., 1996, 2006). Ratiometric imaging of fluorescent protein–fused Akt-PH or Btk-PH domains, which are fluorescent probes for PI(3,4,5)P_3_, relative to the plasma membrane marker has demonstrated that levels of PI(3,4,5)P_3_ are markedly increased after circular ruffle formation (Araki et al., 2007; Yoshida et al., 2009; Welliver and Swanson, 2012). The morphological features of cup-shaped circular ruffles restrict the diffusion of membrane proteins and further amplify local signaling inside the cups (Welliver et al., 2011). PI(3,4,5)P_3_ binds to the PH domains of several proteins, such as Akt, Btk, PDK1, and ARNO, and subsequently leads to their downstream signaling pathways. Unlike PI(4,5)P_2_, PI(3,4,5)P_3_ is not required for actin polymerization but instead contributes to actin depolymerization and remodeling through activating ADF/cofilin (Rupper et al., 2001; Nishita et al., 2004; Araki et al., 2007). PI(3,4,5)P_3_ is also known to activate Rac1 through recruiting Vav, a Rac1 GEF (Patel et al., 2002).

PI(3,4,5)P_3_ is thought to be converted into PI(3,4)P_2_ by SHIP 5-phosphatase. Indeed, imaging of PI(3,4)P_2_ with the TAPP domain has shown that the timing of PI(3,4)P_2_ formation is almost simultaneous with the disappearance of PI(3,4,5)P_3_ (Welliver and Swanson, 2012).

### Class III phosphatidylinositol 3-kinase (PI3K) in macropinosome maturation

A few minutes after macropinosome formation, a considerable amount of PI(3)P is found on the macropinosome membrane. Because PI(3)P production is inhibited by 3-methyladenine, a class III PI3K inhibitor, class III PI3K primarily accounts for the PI(3)P production on macropinosomes. We have reported that the class III PI3K inhibitor prevents the recruitment of EEA1, a PI(3)P-binding membrane tethering protein, to macropinosomes and blocks the homotypic fusion of early macropinosomes (Hamasaki et al., 2004; Araki et al., 2006). Live-cell imaging of PI(3)P with the fluorescent protein-fused FYVE domain demonstrates that PI(3)P is transiently found on the membranes of Rab5-positive early macropinosomes in macrophages (Yoshida et al., 2009); however, in EGF-induced macropinosomes in A431 cells, PI(3)P remains on the membranes as long as macropinosomes are present (Araki et al., 2006). Unlike macrophage macropinosomes, macropinosomes in A431 cells never mature to fuse with lysosomes. The conversion of PI(3)P to PI(3,5)P_2_ by PIKfyve activity may be required for further macropinosome maturation to fuse with late endosomes or lysosomes in the degradation pathway (Kerr et al., 2010). Sorting nexin 5 (SNX5), which possesses a PX domain that binds PI(3)P, is localized on EEA1-positive macropinosomes and tubular extensions from these macropinosomes (Kerr et al., 2006; Lim et al., 2008; Kerr and Teasdale, 2009). The dissociation of SNX5-positive tubules from the macropinosome is thought to promote its maturation.

### Degradation of PI(3,4,5)P_3_ in membrane ruffles

In addition to the burst of PI(3,4,5)P_3_ production inside of circular ruffles (macropinocytic cups), PI(3,4,5)P_3_ is produced and degraded to a lesser extent in the dorsal membrane ruffles. Using fluid-phase uptake mutants of *C. elegans*, we have identified myotubularin-related proteins (MTMRs) as phosphoinositide 3-phosphatases that function at membrane ruffles (Maekawa et al., 2014). In the next two subsections, we describe the usefulness of *C. elegans* mutants in identifying important genes for macropinocytosis, as well as the sequential breakdown of PI(3,4,5)P_3_→PI(3,4)P_2_ →PI(3)P→PI in the ruffling membrane through the action of SHIP 5-phosphatase, inositol polyphosphate 4-phosphatase (INPP4), and myotubularin-related proteins (MTMRs).

#### Fluid-phase uptake mutants in C. elegans

**Figure 3:**
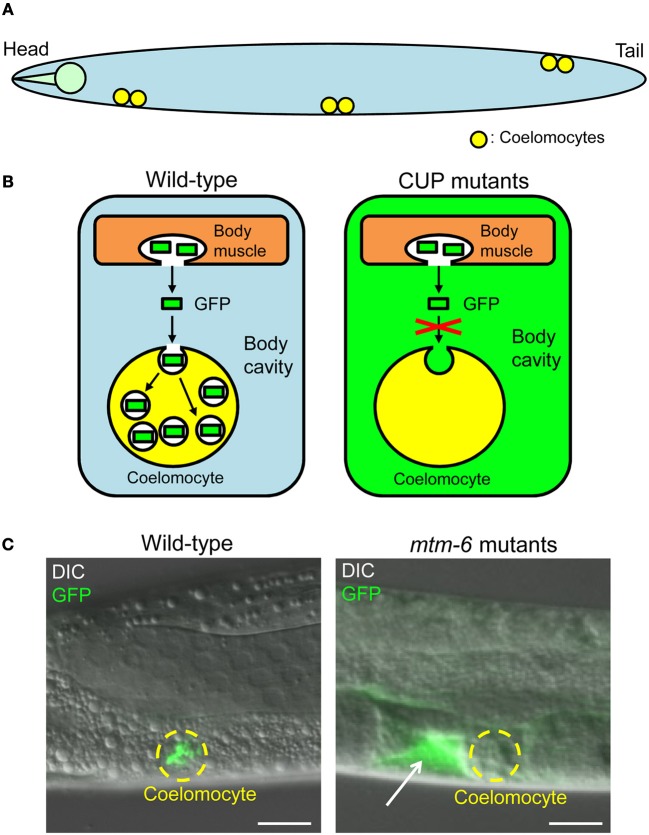
**Fluid-phase uptake in coelomocytes of *Caenorhabditis elegans*. (A)** *Caenorhabditis elegans* generally possesses six coelomocytes (indicated by yellow) in its body cavity. This simple diagram of *C. elegans* is drawn from the side of the organism. Note that the other tissues of *C. elegans* are not shown for the sake of explanation. **(B)** Diagrams of the fluid-phase endocytosis assay. In wild-type worms containing *myo-3p*::ssGFP, GFP synthesized in the body wall muscle cells is secreted into the body cavity and taken up by coelomocytes. In wild-type worms, GFP signals are detected only in the coelomocytes. In CUP mutants containing *myo-3p*::ssGFP, GFP is not endocytosed by coelomocytes and is therefore accumulated in the body cavity. Note that the coelomocytes are magnified for the sake of explanation. **(C)** Images of wild-type worms and *mtm-6*(*ok330)III* mutants (one of the CUP mutants; see Supplementary Table 1). All worms contain *myo-3p*::ssGFP. Coelomocytes in the *mtm-6* mutants do not have GFP-positive vesicles, and GFP accumulates in the body cavity (indicated by a white arrow). Coelomocytes are indicated by broken yellow lines. Scale bars: 10 μm (adapted from Maekawa et al., 2014).

*C. elegans* contains six scavenger-like cells, called coelomocytes, in its pseudocoelom (body cavity) (Figure 3A). Coelomocytes actively and continuously endocytose fluids and solutes to clear them from the body cavity. Considering these coelomocyte characteristics, Fares and Greenwald developed an elegant assay to monitor the endocytic activity of coelomocytes *in situ* (Figure 3B) (Fares and Greenwald, 2001). This assay employs a transgenic worm *(arIs37)* containing *myo-3p::ssGFP*. The transgene induces the synthesis of a secretory signal sequence–GFP chimera in the body wall muscles. GFP is secreted into the body cavity from the body wall muscles and then endocytosed by coelomocytes. GFP is thus only detected in the coelomocytes of the transgenic worms (Figures 3B,C). Through examining known viable endocytosis mutants and RNAi results for other known endocytosis genes, possible causal genes for *c*oelomocyte *up*take defective (CUP) phenotypes were determined. In these CUP mutants, GFP accumulates in the body cavity because of the defective uptake of GFP by coelomocytes (Figures 3B,C). The CUP genes identified to date are listed in Supplementary Table 1.

#### Phosphatases that degrade 3-phosphorylated phosphoinositides in membrane ruffles

Macropinocytosis in mammalian cells may resemble the process of fluid uptake in coelomocytes. We therefore examined if mammalian orthologs of the CUP genes are involved in macropinocytosis. We found that myotubularin-related protein 6 (MTMR6) and myotubularin-related protein 9 (MTMR9), mammalian ortholog of *mtm-6* and *mtm-9*, are essential for macropinocytosis in A431 cells (Maekawa et al., 2014). MTMR6 and MTMR9 belong to the myotubularin family phosphoinositide 3-phosphatases (Robinson and Dixon, 2006; Hnia et al., 2012), suggesting that the degradation of PI(3)P to PI is required for macropinosome formation. Cells depleted of MTMR6 or MTMR9 still show membrane ruffling after EGF stimulation, suggesting that MTMR6 and MTMR9 function in the steps that follow the formation of membrane ruffles (Maekawa et al., 2014).

PI(3,4,5)P_3_ and PI(3,4)P_2_ are transiently generated in dorsal membrane ruffles during circular ruffle formation in PDGF-stimulated NIH3T3 cells (Hasegawa et al., 2011). As MTMR6 is a 3-phosphatase (Schaletzky et al., 2003), we reasoned that PI(3,4)P_2_ is dephosphorylated by 4-phosphatase to provide the substrate PI(3)P to MTMR6. INPP4B is a specific 4-phosphatase of PI(3,4)P_2_ (Norris et al., 1997; Ivetac et al., 2005; Gewinner et al., 2009). Using RNAi knockdown experiments, we found that INPP4B is essential for macropinocytosis in EGF-stimulated A431 cells (Maekawa et al., 2014). Similarly to the knockdowns of MTMR6 and MTMR9, the knockdown of INPP4B did not inhibit EGF-induced membrane ruffling. Therefore, INPP4B likely also functions at the steps after the formation of membrane ruffles.

In light of a report that SHIP2, a 5-phosphatase of PI(3,4,5)P_3_, is involved in PDGF-induced circular ruffle formation in NIH3T3 cells after PDGF (Hasegawa et al., 2011), these results suggest that PI(3,4,5)P_3_ is sequentially converted to PI in membrane ruffles by specific PI phosphatases and that this process is indispensable for circular ruffle/macropinocytic cup formation. Live cell imaging of EGF-stimulated A431 cells expressing fluorescent Akt-PH or TAPP1-PH domains has consistently detected significant levels of PI(3,4,5)_3_ and PI(3,4)P_2_ at the membrane of EGF-stimulated ruffling (Maekawa et al., 2014), although these increased levels are not as high as those detected on the membrane inside of macropinocytic cups after circular ruffle formation (Welliver and Swanson, 2012).

When monitoring PI(3)P using a GFP-fused FYVE domain, PI(3)P is faintly observed in the ruffling membrane. However, in MTMR6-depeleted A431 cells with RNAi, significant levels of PI(3)P are detected in the membrane of EGF-induced dorsal ruffling. Double knockdown of MTMR6 and SHIP2 or of MTMR6 and INPP4B prevents the emergence of PI(3)P after EGF stimulation, indicating that the PI(3)P in the membrane ruffles is produced through the sequential breakdown of PI(3,4,5)P_3_ and PI(3,4)P_2_ by SHIP2 and INPP4B. The level of PI(3)P in the membrane ruffle is much lower than that in the membranes of early macropinosomes, which is produced through the phosphorylation of PI by class III PI3K. However, the transient PI(3)P production in the membrane ruffle may be required for the activation of KCa3.1, a Ca^2+^-activated K^+^ channel (Srivastava et al., 2005). Actually, KCa3.1 localizes to membrane ruffles and is essential for macropinosome formation (Maekawa et al., 2014).

## Macropinocytosis in *dictyostelium discoideum*

*Dictyostelium discoideum* is a genetically and biochemically tractable soil ameba. In *D. discoideum*, active macropinocytosis constitutively occurs for the uptake of nutrients from the environment and can be experimentally detected through the observation of macropinosomes labeled by fluorophore-conjugated dextran, a fluid-phase marker, added to the medium. Therefore, the molecular mechanisms of macropinocytosis in *D. discoideum* have been elucidated, as have those of phagocytosis and chemotaxis, using gene disruption and protein expression (Maniak, 2001). Similarly to that in mammalian cells, macropinocytosis in *D. discoideum* is actin-dependent (Hacker et al., 1997). Null mutants of coronin, an actin-binding protein, and Daip1, an actin-interacting protein, present defective macropinocytosis (Maniak et al., 1995; Konzok et al., 1999). Rac1 GTPase also regulates macropinocytosis in *D. discoideum* (Dumontier et al., 2000). Another study using null mutants and an inhibitor has indicated that class I PI3K and its downstream effector, protein kinase B (PKB/Akt), regulate macropinocytosis in this species (Rupper et al., 2001). More recently, Hoeller et al. (2013) revealed two distinct functions for class I PI3K isoforms in macropinocytosis using systematic genetic ablation. PI3K1 and 2 produce patches of PI(3,4,5)P_3_ associated with actin-dependent ruffle formation, and PI3K4 is required for the conversion of ruffles into intracellular macropinosomes. Furthermore, these authors identified two specific Ras proteins, RasG and RasS, that interact with PI3K1/2 and PI3K4 (Hoeller et al., 2013).

Through expressing probes for phosphoinositides, the dynamics of 3-phosphoinositides can be visualized during macropinocytosis in *D. discoideum* (Dormann et al., 2004; Hoeller et al., 2013; Veltman et al., 2014). PI(3,4,5)P_3_ transiently increases at the macropinocytic cups, followed by rapid PI(3,4)P_2_ generation at the periphery of the macropinosomes upon internalization, suggesting that PI(3,4,5)P_3_ is degraded to PI(3,4)P_2_ through macropinosome maturation (Dormann et al., 2004). Similar phosphoinositide kinetics are observed during macropinocytosis in mammalian macrophages (Welliver and Swanson, 2012). Thus, this free-living ameba shares many features of macropinocytosis with mammalian phagocytes such as macrophages (Cardelli, 2001). Although careful attention should be given to the differences in the molecular mechanisms of macropinocytosis between *D. discoideum* and mammalian cells, information from this species is generally helpful for the analysis of mammalian macropinocytosis.

## Concluding remarks

Our current knowledge of the macropinocytic pathway in mammals has been primarily derived from pharmacological and gene overexpression approaches. However, due to the off-target effects or incomplete inhibition, it is occasionally difficult to identify a single key gene for a certain process of macropinocytosis. In this respect, genetic studies of *C. elegans* and *D. discoideum* are useful for identifying crucial regulators of macropinocytosis, although careful attention must be given to the differences in the molecular mechanisms among different organisms. Additionally, the application of newly developed genome editing systems such as CRISPR/Cas9 may shed light on this endocytic mechanism in mammalian cells. To better understand the dynamic mechanisms coordinating macropinosome formation and maturation in both space and time, both the genetic identification of key molecules and live cell imaging are crucial. Combining morphological studies with FRET imaging, super-resolution microscopy, and/or optogenetics will be quite valuable for dissecting the dynamic molecular mechanisms of this process.

Although still incompletely understood, the molecular mechanism that controls macropinocytosis has been greatly elucidated in recent years. It is now apparent that large numbers of signaling and mechanistic molecules spatiotemporally regulate the coordinated processes of macropinosome formation and maturation. The complex interplay of small GTPase molecular switches and phosphoinositides orchestrates the highly sophisticated processes of macropinocytosis (Figure 1). However, the regulation of macropinocytosis may partly be dependent on cell type. Macropinocytosis is constitutive in antigen-presenting cells such as macrophages and dendritic cells (Steinman and Swanson, 1995; Swanson and Watts, 1995), but it can also be rapidly induced by growth factors in other cell types, such as epithelial cells and fibroblasts. Macropinosomes usually mature and merge with the lysosomal degradative pathway. However, macropinosomes in certain cell types, such as A431 cells, do not deliver their fluid contents to the degradation pathway and are instead extracellularly regurgitated by recycling pathways (Hewlett, 1994; Hamasaki et al., 2004). Although the biological significance of this backflow is unknown, it is of special interest for elucidating the differences in the molecular mechanisms underlying the distinct emergence and fate of macropinosomes in different cell types. If we can determine these differences using molecular and signaling comparisons of the two distinct macropinocytic pathways, we can manipulate macropinocytosis through activating or inhibiting the key signals. Several pathogenic microorganisms that enter thorough macropinocytosis interfere with macropinosome maturation to survive inside of the cell. The manipulation of macropinocytosis would therefore also be to our advantage in developing therapeutic strategies for human diseases involve macropinocytic activity.

### Conflict of interest statement

The authors declare that the research was conducted in the absence of any commercial or financial relationships that could be construed as a potential conflict of interest.

## References

[B1] Alpuche-Aranda C. M. Berthiaume E. P. Mock B. Swanson J. A. Miller S. I. (1995). Spacious phagosome formation within mouse macrophages correlates with Salmonella serotype pathogenicity and host susceptibility. Infect. Immun. 63, 4456–4462 7591085 10.1128/iai.63.11.4456-4462.1995 PMC173634

[B2] Amyere M. Payrastre B. Krause U. Van Der Smissen P. Veithen A. Courtoy P. J. (2000). Constitutive macropinocytosis in oncogene-transformed fibroblasts depends on sequential permanent activation of phosphoinositide 3-kinase and phospholipase C. Mol. Biol. Cell 11, 3453–3467 10.1091/mbc.11.10.3453 11029048 PMC15006

[B3] Araki N. Egami Y. Watanabe Y. Hatae T. (2007). Phosphoinositide metabolism during membrane ruffling and macropinosome formation in EGF-stimulated A431 cells. Exp. Cell Res. 313, 1496–1507 10.1016/j.yexcr.2007.02.012 17368443

[B4] Araki N. Hamasaki M. Egami Y. Hatae T. (2006). Effect of 3-methyladenine on the fusion process of macropinosomes in EGF-stimulated A431 Cells. Cell Struct. Funct. 31, 145–157 10.1247/csf.06029 17146146

[B5] Araki N. Hatae T. Yamada T. Hirohashi S. (2000). Actinin-4 is preferentially involved in circular ruffling and macropinocytosis in mouse macrophages: analysis by fluorescence ratio imaging. J. Cell Sci. 113, 3329–3340 10954430 10.1242/jcs.113.18.3329

[B6] Araki N. Ikeda Y. Kato T. Kawai K. Egami Y. Miyake K. (2014). Development of an automated fluorescence microscopy system for photomanipulation of genetically encoded photoactivatable proteins (optogenetics) in live cells. Microscopy 63, 255–260 10.1093/jmicro/dfu003 24523516

[B7] Araki N. Johnson M. T. Swanson J. A. (1996). A role for phosphoinositide 3-kinase in the completion of macropinocytosis and phagocytosis by macrophages. J. Cell Biol. 135, 1249–1260 10.1083/jcb.135.5.1249 8947549 PMC2121091

[B8] Balañá M. E. Niedergang F. Subtil A. Alcover A. Chavrier P. Dautry-Varsat A. (2005). ARF6 GTPase controls bacterial invasion by actin remodelling. J. Cell Sci. 118, 2201–2210 10.1242/jcs.02351 15897187

[B9] Bar-Sagi D. Feramisco J. R. (1986). Induction of membrane ruffling and fluid-phase pinocytosis in quiescent fibroblasts by ras proteins. Science 233, 1061–1068 10.1126/science.3090687 3090687

[B10] Cardelli J. (2001). Phagocytosis and macropinocytosis in Dictyostelium: phosphoinositide-based processes, biochemically distinct. Traffic 2, 311–320 10.1034/j.1600-0854.2001.002005311.x 11350627

[B11] Colicelli J. (2004). Human RAS superfamily proteins and related GTPases. Sci. STKE 2004:RE13 10.1126/stke.2502004re13 15367757 PMC2828947

[B12] Commisso C. Davidson S. M. Soydaner-Azeloglu R. G. Parker S. J. Kamphorst J. J. Hackett S. (2013). Macropinocytosis of protein is an amino acid supply route in Ras-transformed cells. Nature 497, 633–637 10.1038/nature12138 23665962 PMC3810415

[B13] Coyne C. B. Shen L. Turner J. R. Bergelson J. M. (2007). Coxsackievirus entry across epithelial tight junctions requires occludin and the small GTPases Rab34 and Rab5. Cell Host Microbe 2, 181–192 10.1016/j.chom.2007.07.003 18005733 PMC2719558

[B14] Dharmawardhane S. Schurmann A. Sells M. A. Chernoff J. Schmid S. L. Bokoch G. M. (2000). Regulation of macropinocytosis by p21-activated kinase-1. Mol. Biol. Cell 11, 3341–3352 10.1091/mbc.11.10.3341 11029040 PMC14996

[B15] Di Paolo G. De Camilli P. (2006). Phosphoinositides in cell regulation and membrane dynamics. Nature 443, 651–657 10.1038/nature05185 17035995

[B16] Dormann D. Weijer G. Dowler S. Weijer C. J. (2004). *In vivo* analysis of 3-phosphoinositide dynamics during Dictyostelium phagocytosis and chemotaxis. J. Cell Sci. 117, 6497–6509 10.1242/jcs.01579 15572406

[B17] Dumontier M. Höcht P. Mintert U. Faix J. (2000). Rac1 GTPases control filopodia formation, cell motility, endocytosis, cytokinesis and development in Dictyostelium. J. Cell Sci. 113, 2253–2265 10825297 10.1242/jcs.113.12.2253

[B18] Egami Y. Araki N. (2009). Dynamic changes in the spatiotemporal localization of Rab21 in live RAW264 cells during macropinocytosis. PLoS ONE 4:e6689 10.1371/journal.pone.0006689 19693279 PMC2726762

[B19] Egami Y. Araki N. (2012). Spatiotemporal localization of Rab20 in live RAW264 macrophages during macropinocytosis. Acta Histochem. Cytochem. 45, 317–323 10.1267/ahc.12014 23378675 PMC3554782

[B20] Ellerbroek S. M. Wennerberg K. Arthur W. T. Dunty J. M. Bowman D. R. DeMali K. A. (2004). SGEF, a RhoG guanine nucleotide exchange factor that stimulates macropinocytosis. Mol. Biol. Cell 15, 3309–3319 10.1091/mbc.E04-02-0146 15133129 PMC452585

[B21] Fares H. Greenwald I. (2001). Genetic analysis of endocytosis in *Caenorhabditis elegans*: coelomocyte uptake defective mutants. Genetics 159, 133–145 11560892 10.1093/genetics/159.1.133 PMC1461804

[B22] Feliciano W. D. Yoshida S. Straight S. W. Swanson J. A. (2011). Coordination of the Rab5 cycle on macropinosomes. Traffic 12, 1911–1922 10.1111/j.1600-0854.2011.01280.x 21910808 PMC3213305

[B23] Fernandez-Borja M. Janssen L. Verwoerd D. Hordijk P. Neefjes J. (2005). RhoB regulates endosome transport by promoting actin assembly on endosomal membranes through Dia1. J. Cell Sci. 118, 2661–2670 10.1242/jcs.02384 15944396

[B24] Fujii M. Kawai K. Egami Y. Araki N. (2013). Dissecting the roles of Rac1 activation and deactivation in macropinocytosis using microscopic photo-manipulation. Sci. Rep. 3:2385 10.1038/srep02385 23924974 PMC3737501

[B25] Garcia-del Portillo F. Finlay B. B. (1994). Salmonella invasion of nonphagocytic cells induces formation of macropinosomes in the host cell. Infect. Immun. 62, 4641–4645 7927733 10.1128/iai.62.10.4641-4645.1994 PMC303156

[B26] Gewinner C. Wang Z. C. Richardson A. Teruya-Feldstein J. Etemadmoghadam D. Bowtell D. (2009). Evidence that inositol polyphosphate 4-phosphatase type II is a tumor suppressor that inhibits PI3K signaling. Cancer Cell 16, 115–125 10.1016/j.ccr.2009.06.006 19647222 PMC2957372

[B27] Grimmer S. van Deurs B. Sandvig K. (2002). Membrane ruffling and macropinocytosis in A431 cells require cholesterol. J. Cell Sci. 115, 2953–2962 12082155 10.1242/jcs.115.14.2953

[B28] Hacker U. Albrecht R. Maniak M. (1997). Fluid-phase uptake by macropinocytosis in Dictyostelium. J. Cell Sci. 110, 105–112 9044041 10.1242/jcs.110.2.105

[B29] Haga Y. Miwa N. Jahangeer S. Okada T. Nakamura S. (2009). CtBP1/BARS is an activator of phospholipase D1 necessary for agonist-induced macropinocytosis. EMBO J. 28, 1197–1207 10.1038/emboj.2009.78 19322195 PMC2664659

[B30] Hall A. (1998). Rho GTPases and the actin cytoskeleton. Science 279, 509–514 10.1126/science.279.5350.509 9438836

[B31] Hall A. Nobes C. D. (2000). Rho GTPases: molecular switches that control the organization and dynamics of the actin cytoskeleton. Philos. Trans. R. Soc. Lond. B Biol. Sci. 355, 965–970 10.1098/rstb.2000.0632 11128990 PMC1692798

[B32] Hamasaki M. Araki N. Hatae T. (2004). Association of early endosomal autoantigen 1 with macropinocytosis in EGF-stimulated A431 cells. Anat. Rec. A Discov. Mol. Cell. Evol. Biol. 277, 298–306 10.1002/ar.a.20027 15052657

[B33] Hasegawa J. Tokuda E. Tenno T. Tsujita K. Sawai H. Hiroaki H. (2011). SH3YL1 regulates dorsal ruffle formation by a novel phosphoinositide-binding domain. J. Cell Biol. 193, 901–916 10.1083/jcb.201012161 21624956 PMC3105542

[B34] Hewlett L. J. (1994). The coated pit and macropinocytic pathways serve distinct endosome populations. J. Cell Biol. 124, 689–703 10.1083/jcb.124.5.689 8120092 PMC2119947

[B35] Hnia K. Vaccari I. Bolino A. Laporte J. (2012). Myotubularin phosphoinositide phosphatases: cellular functions and disease pathophysiology. Trends Mol. Med. 18, 317–327 10.1016/j.molmed.2012.04.004 22578719

[B36] Hoeller O. Bolourani P. Clark J. Stephens L. R. Hawkins P. T. Weiner O. D. (2013). Two distinct functions for PI3-kinases in macropinocytosis. J. Cell Sci. 126, 4296–4307 10.1242/jcs.134015 23843627 PMC3772393

[B37] Honda A. Nogami M. Yokozeki T. Yamazaki M. Nakamura H. Watanabe H. (1999). Phosphatidylinositol 4-phosphate 5-kinase alpha is a downstream effector of the small G protein ARF6 in membrane ruffle formation. Cell 99, 521–532 10.1016/S0092-8674(00)81540-8 10589680

[B38] Humphreys D. Davidson A. C. Hume P. J. Makin L. E. Koronakis V. (2013). Arf6 coordinates actin assembly through the WAVE complex, a mechanism usurped by Salmonella to invade host cells. Proc. Natl. Acad. Sci. U.S.A. 110, 16880–16885 10.1073/pnas.1311680110 24085844 PMC3801044

[B39] Ivetac I. Munday A. D. Kisseleva M. V. Zhang X.-M. Luff S. Tiganis T. (2005). The type Ialpha inositol polyphosphate 4-phosphatase generates and terminates phosphoinositide 3-kinase signals on endosomes and the plasma membrane. Mol. Biol. Cell 16, 2218–2233 10.1091/mbc.E04-09-0799 15716355 PMC1087230

[B40] Kabayama H. Nakamura T. Takeuchi M. Iwasaki H. Taniguchi M. Tokushige N. (2009). Ca^2+^ induces macropinocytosis via F-actin depolymerization during growth cone collapse. Mol. Cell. Neurosci. 40, 27–38 10.1016/j.mcn.2008.08.009 18848894

[B41] Kabayama H. Takeuchi M. Taniguchi M. Tokushige N. Kozaki S. Mizutani A. (2011). Syntaxin 1B suppresses macropinocytosis and semaphorin 3A-induced growth cone collapse. J. Neurosci. 31, 7357–7364 10.1523/JNEUROSCI.2718-10.2011 21593320 PMC6622584

[B42] Kerr M. C. Lindsay M. R. Luetterforst R. Hamilton N. Simpson F. Parton R. G. (2006). Visualisation of macropinosome maturation by the recruitment of sorting nexins. J. Cell Sci. 119, 3967–3980 10.1242/jcs.03167 16968745

[B43] Kerr M. C. Teasdale R. D. (2009). Defining macropinocytosis. Traffic 10, 364–371 10.1111/j.1600-0854.2009.00878.x 19192253

[B44] Kerr M. C. Wang J. T. H. Castro N. A. Hamilton N. A. Town L. Brown D. L. (2010). Inhibition of the PtdIns(5) kinase PIKfyve disrupts intracellular replication of Salmonella. EMBO J. 29, 1331–1347 10.1038/emboj.2010.28 20300065 PMC2868569

[B45] Konzok A. Weber I. Simmeth E. Hacker U. Maniak M. Müller-Taubenberger A. (1999). DAip1, a Dictyostelium homologue of the yeast actin-interacting protein 1, is involved in endocytosis, cytokinesis, and motility. J. Cell Biol. 146, 453–464 10.1083/jcb.146.2.453 10427097 PMC2156175

[B46] Kruth H. S. Jones N. L. Huang W. Zhao B. Ishii I. Chang J. (2005). Macropinocytosis is the endocytic pathway that mediates macrophage foam cell formation with native low density lipoprotein. J. Biol. Chem. 280, 2352–2360 10.1074/jbc.M407167200 15533943

[B47] Lanzetti L. Palamidessi A. Areces L. Scita G. Di Fiore P. P. (2004). Rab5 is a signalling GTPase involved in actin remodelling by receptor tyrosine kinases. Nature 429, 309–314 10.1038/nature02542 15152255

[B48] Lewis W. H. (1931). Pinocytosis. Bull. Johns Hopkins Hosp. 49, 17–26

[B49] Liberali P. Kakkonen E. Turacchio G. Valente C. Spaar A. Perinetti G. (2008). The closure of Pak1-dependent macropinosomes requires the phosphorylation of CtBP1/BARS. EMBO J. 27, 970–981 10.1038/emboj.2008.59 18354494 PMC2323256

[B50] Lim J. P. Gleeson P. A. (2011). Macropinocytosis: an endocytic pathway for internalising large gulps. Immunol. Cell Biol. 89, 836–843 10.1038/icb.2011.20 21423264

[B51] Lim J. P. Wang J. T. H. Kerr M. C. Teasdale R. D. Gleeson P. A. (2008). A role for SNX5 in the regulation of macropinocytosis. BMC Cell Biol. 9:58 10.1186/1471-2121-9-58 18854019 PMC2576169

[B52] Maekawa M. Terasaka S. Mochizuki Y. Kawai K. Ikeda Y. Araki N. (2014). Sequential breakdown of 3-phosphorylated phosphoinositides is essential for the completion of macropinocytosis. Proc. Natl. Acad. Sci. U.S.A. 111, E978–E987 10.1073/pnas.1311029111 24591580 PMC3964036

[B53] Maniak M. (2001). Fluid-phase uptake and transit in axenic Dictyostelium cells. Biochim. Biophys. Acta 1525, 197–204 10.1016/S0304-4165(01)00105-2 11257433

[B54] Maniak M. Rauchenberger R. Albrecht R. Murphy J. Gerisch G. (1995). Coronin involved in phagocytosis: dynamics of particle-induced relocalization visualized by a green fluorescent protein tag. Cell 83, 915–924 10.1016/0092-8674(95)90207-4 8521515

[B55] Mercer J. Helenius A. (2009). Virus entry by macropinocytosis. Nat. Cell Biol. 11, 510–520 10.1038/ncb0509-510 19404330

[B56] Mercer J. Helenius A. (2012). Gulping rather than sipping: macropinocytosis as a way of virus entry. Curr. Opin. Microbiol. 15, 490–499 10.1016/j.mib.2012.05.016 22749376

[B57] Nishita M. Wang Y. Tomizawa C. Suzuki A. Niwa R. Uemura T. (2004). Phosphoinositide 3-kinase-mediated activation of cofilin phosphatase Slingshot and its role for insulin-induced membrane protrusion. J. Biol. Chem. 279, 7193–7198 10.1074/jbc.M312591200 14645219

[B58] Nobes C. D. Hall A. (1995). Rho, Rac, and Cdc42 GTPases regulate the assembly of multimolecular focal complexes associated with actin stress fibers, lamellipodia, and filopodia. Cell 81, 53–62 10.1016/0092-8674(95)90370-4 7536630

[B59] Norris F. A. Atkins R. C. Majerus P. W. (1997). The cDNA cloning and characterization of inositol polyphosphate 4-phosphatase type II. Evidence for conserved alternative splicing in the 4-phosphatase family. J. Biol. Chem. 272, 23859–23864 10.1074/jbc.272.38.23859 9295334

[B60] Patel J. C. Galán J. E. (2006). Differential activation and function of Rho GTPases during Salmonella-host cell interactions. J. Cell Biol. 175, 453–463 10.1083/jcb.200605144 17074883 PMC2064522

[B61] Patel J. C. Hall A. Caron E. (2002). Vav regulates activation of Rac but not Cdc42 during FcgammaR-mediated phagocytosis. Mol. Biol. Cell 13, 1215–1226 10.1091/mbc.02-01-0002 11950933 PMC102263

[B62] Pertz O. Hodgson L. Klemke R. L. Hahn K. M. (2006). Spatiotemporal dynamics of RhoA activity in migrating cells. Nature 440, 1069–1072 10.1038/nature04665 16547516

[B63] Porat-Shliom N. Kloog Y. Donaldson J. G. (2008). A unique platform for H-Ras signaling involving clathrin-independent endocytosis. Mol. Biol. Cell 19, 765–775 10.1091/mbc.E07-08-0841 18094044 PMC2262976

[B64] Racoosin E. L. Swanson J. A. (1993). Macropinosome maturation and fusion with tubular lysosomes in macrophages. J. Cell Biol. 121, 1011–1020 10.1083/jcb.121.5.1011 8099075 PMC2119679

[B65] Radhakrishna H. Al-Awar O. Khachikian Z. Donaldson J. G. (1999). ARF6 requirement for Rac ruffling suggests a role for membrane trafficking in cortical actin rearrangements. J. Cell Sci. 112, 855–866 10036235 10.1242/jcs.112.6.855

[B66] Radhakrishna H. Klausner R. D. Donaldson J. G. (1996). Aluminum fluoride stimulates surface protrusions in cells overexpressing the ARF6 GTPase. J. Cell Biol. 134, 935–947 10.1083/jcb.134.4.935 8769418 PMC2120964

[B67] Ridley A. J. (2006). Rho GTPases and actin dynamics in membrane protrusions and vesicle trafficking. Trends Cell Biol. 16, 522–529 10.1016/j.tcb.2006.08.006 16949823

[B68] Robinson F. L. Dixon J. E. (2006). Myotubularin phosphatases: policing 3-phosphoinositides. Trends Cell Biol. 16, 403–412 10.1016/j.tcb.2006.06.001 16828287

[B69] Rupper A. Lee K. Knecht D. Cardelli J. (2001). Sequential activities of phosphoinositide 3-kinase, PKB/Aakt, and Rab7 during macropinosome formation in Dictyostelium. Mol. Biol. Cell 12, 2813–2824 10.1091/mbc.12.9.2813 11553719 PMC59715

[B70] Sallusto F. Cella M. Danieli C. Lanzavecchia A. (1995). Dendritic cells use macropinocytosis and the mannose receptor to concentrate macromolecules in the major histocompatibility complex class II compartment: downregulation by cytokines and bacterial products. J. Exp. Med. 182, 389–400 10.1084/jem.182.2.389 7629501 PMC2192110

[B71] Samson T. Welch C. Monaghan-Benson E. Hahn K. M. Burridge K. (2010). Endogenous RhoG is rapidly activated after epidermal growth factor stimulation through multiple guanine-nucleotide exchange factors. Mol. Biol. Cell 21, 1629–1642 10.1091/mbc.E09-09-0809 20237158 PMC2861620

[B72] Schafer D. A. D'Souza-Schorey C. Cooper J. A. (2000). Actin assembly at membranes controlled by ARF6. Traffic 1, 896–907 10.1034/j.1600-0854.2000.011108.x 11273133

[B73] Schaletzky J. Dove S. K. Short B. Lorenzo O. Clague M. J. Barr F. A. (2003). Phosphatidylinositol-5-phosphate activation and conserved substrate specificity of the myotubularin phosphatidylinositol 3-phosphatases. Curr. Biol. 13, 504–509 10.1016/S0960-9822(03)00132-5 12646134

[B74] Schnatwinkel C. Christoforidis S. Lindsay M. R. Uttenweiler-Joseph S. Wilm M. Parton R. G. (2004). The Rab5 effector Rabankyrin-5 regulates and coordinates different endocytic mechanisms. PLoS Biol. 2:E261 10.1371/journal.pbio.0020261 15328530 PMC514490

[B75] Schwartz S. L. Cao C. Pylypenko O. Rak A. Wandinger-Ness A. (2007). Rab GTPases at a glance. J. Cell Sci. 120, 3905–3910 10.1242/jcs.015909 17989088

[B76] Scott C. C. Dobson W. Botelho R. J. Coady-Osberg N. Chavrier P. Knecht D. A. (2005). Phosphatidylinositol-4,5-bisphosphate hydrolysis directs actin remodeling during phagocytosis. J. Cell Biol. 169, 139–149 10.1083/jcb.200412162 15809313 PMC2171893

[B77] Somsel Rodman J. Wandinger-Ness A. (2000). Rab GTPases coordinate endocytosis. J. Cell Sci. 113, 183–192 10633070 10.1242/jcs.113.2.183

[B78] Srivastava S. Li Z. Lin L. Liu G. Ko K. Coetzee W. A. (2005). The phosphatidylinositol 3-phosphate phosphatase myotubularin- related protein 6 (MTMR6) is a negative regulator of the Ca^2+^-activated K^+^ channel KCa3.1. Mol. Cell. Biol. 25, 3630–3638 10.1128/MCB.25.9.3630-3638.2005 15831468 PMC1084293

[B79] Steinman R. M. Swanson J. (1995). The endocytic activity of dendritic cells. J. Exp. Med. 182, 283–288 10.1084/jem.182.2.283 7629494 PMC2192115

[B80] Sun P. Yamamoto H. Suetsugu S. Miki H. Takenawa T. Endo T. (2003). Small GTPase Rah/Rab34 is associated with membrane ruffles and macropinosomes and promotes macropinosome formation. J. Biol. Chem. 278, 4063–4071 10.1074/jbc.M208699200 12446704

[B81] Swanson J. A. (1989). Phorbol esters stimulate macropinocytosis and solute flow through macrophages. J. Cell Sci. 94, 135–142 2613767 10.1242/jcs.94.1.135

[B82] Swanson J. A. (2008). Shaping cups into phagosomes and macropinosomes. Nat. Rev. Mol. Cell Biol. 9, 639–649 10.1038/nrm2447 18612320 PMC2851551

[B83] Swanson J. A. Watts C. (1995). Macropinocytosis. Trends Cell Biol. 5, 424–428 10.1016/S0962-8924(00)89101-1 14732047

[B84] Terebiznik M. R. Vieira O. V. Marcus S. L. Slade A. Yip C. M. Trimble W. S. (2002). Elimination of host cell PtdIns(4,5)P_2_ by bacterial SigD promotes membrane fission during invasion by *Salmonella*. Nat. Cell Biol. 4, 766–773 10.1038/ncb854 12360287

[B85] Veltman D. M. Lemieux M. G. Knecht D. A. Insall R. H. (2014). PIP3-dependent macropinocytosis is incompatible with chemotaxis. J. Cell Biol. 204, 497–505 10.1083/jcb.201309081 24535823 PMC3926956

[B86] Welliver T. P. Chang S. L. Linderman J. J. Swanson J. A. (2011). Ruffles limit diffusion in the plasma membrane during macropinosome formation. J. Cell Sci. 124, 4106–4114 10.1242/jcs.091538 22194306 PMC3244989

[B87] Welliver T. P. Swanson J. A. (2012). A growth factor signaling cascade confined to circular ruffles in macrophages. Biol. Open 1, 754–760 10.1242/bio.20121784 23213469 PMC3507227

[B88] West M. A. Prescott A. R. Eskelinen E.-L. Ridley A. J. Watts C. (2000). Rac is required for constitutive macropinocytosis by dendritic cells but does not control its downregulation. Curr. Biol. 10, 839–848 10.1016/S0960-9822(00)00595-9 10899002

[B89] Wu Y. I. Frey D. Lungu O. I. Jaehrig A. Schlichting I. Kuhlman B. (2009). A genetically encoded photoactivatable Rac controls the motility of living cells. Nature 461, 104–108 10.1038/nature08241 19693014 PMC2766670

[B90] Yoshida S. Hoppe A. D. Araki N. Swanson J. A. (2009). Sequential signaling in plasma-membrane domains during macropinosome formation in macrophages. J. Cell Sci. 122, 3250–3261 10.1242/jcs.053207 19690049 PMC2736863

[B91] Zawistowski J. S. Sabouri-Ghomi M. Danuser G. Hahn K. M. Hodgson L. (2013). A RhoC biosensor reveals differences in the activation kinetics of RhoA and RhoC in migrating cells. PLoS ONE 8:e79877 10.1371/journal.pone.0079877 24224016 PMC3818223

[B92] Zerial M. McBride H. (2001). Rab proteins as membrane organizers. Nat. Rev. Mol. Cell Biol. 2, 107–117 10.1038/35052055 11252952

[B93] Zhang Q. Calafat J. Janssen H. Greenberg S. (1999). ARF6 is required for growth factor- and rac-mediated membrane ruffling in macrophages at a stage distal to rac membrane targeting. Mol. Cell. Biol. 19, 8158–8168 10567541 10.1128/mcb.19.12.8158 PMC84900

