# Supplementary material for: Small GTPases and phosphoinositides in the regulatory mechanisms of macropinosome formation and maturation
Source: Front Physiol. 2014 Sep 30;5:374. doi: 10.3389/fphys.2014.00374 (PMC4179697; doi:10.3389/fphys.2014.00374)
Supplement: Supplementary file 1 [file DataSheet1.DOCX]

***Supplementary Material***

**Small GTPases and phosphoinositides in the regulatory mechanisms of macropinosome formation and maturation**

Youhei Egami^1^, Tomohiko Taguchi^2,3^, Masashi Maekawa^2,4^, Hiroyuki Arai^2,3^, Nobukazu Araki^1^*

^1^Department of Histology and Cell Biology, School of Medicine, Kagawa University, Miki, Kagawa, Japan

^2^Department of Health Chemistry, Graduate School of Pharmaceutical Sciences, University of Tokyo, Tokyo, Japan

^3^Pathological Cell Biology Laboratory, Graduate School of Pharmaceutical Sciences, University of Tokyo, Tokyo, Japan

^4^ Keenan Research Centre, Li Ka Shing Knowledge Institute, St. Michael’s Hospital,Toronto, ON, M5B1W8, Canada

*** Correspondence:** Prof. Nobukazu Araki

Department of Histology and Cell Biology, School of Medicine, Kagawa University, 1750-1 Ikenobe, Miki, Kagawa 761-0793, Japan

[naraki@med.kagawa-u.ac.jp](mailto:naraki@med.kagawa-u.ac.jp)

1. **Supplementary Table**

**Supplementary Table 1. List of the identified CUP (coelomocyte uptake defective) mutants**

| Systematic name | Standard name | Mammalian homologue | Require for macropinocytosis | Functional descriptions | References |
| --- | --- | --- | --- | --- | --- |
| F25D7.1 | *cup-2* | Derlin1 | n. d. | Component of ERAD | 1, 2 |
| C02C3.3 | *cup-4* | CHRNB1 | n. d. | Cholinergic receptor | 1, 3 |
| F53A2.8 | *mtm-6 (cup-6)* | MTMR6 | yes | Lipid phosphatase | 1, 4 |
| Y39H10A.3 | *mtm-9 (cup-10)* | MTMR9 | yes | Lipid phosphatase | 1, 4 |
| Y67D8C.10 | *mca-3 (cup-7)* | ATP2B4 | n. d. | Calcium transporter | 1, 5 |
| C02C6.1 | *dyn-1** | Dynamin-2 | yes | GTPase for membrane scission | 1 |
| W06B4.3 | *vps-18*** | VPS18 | n. d. | Vacuolar protein sorting | 6 |
| C10G8.6 | *ceh-34**** | SIX2 | n. d. | Homeodomain binding protein | 7 |
| Y42H9AR.3 | *rabs-5* | Rabenosyn-5 | n. d. | Rab5 effector | 8 |
| C44C1.4 | *vps-45* | VPS45 | n. d. | Vacuolar protein sorting | 8 |
| ZK524.2 | *unc-13***** | UNC-13 Homologue | n. d. | Unknown | 9 |
| ZK897.1 | *unc-31***** | CADPS | n. d. | Secretion activator | 9 |
| W06H8.1 | *rme-1* | EHD1 | n. d. | EH domain protein | 1, 10 |
| F46F6.1 | *rme-4* | DENND1/connecdenn | n. d. | GEF for Rab proteins | 11 |
| F49E7.1 | *rme-6* | GAPEX | n. d. | GEF for Rab proteins | 1, 12 |
| F18C12.2 | *rme-8* | DNAJC13 | n. d. | Chaperon cofactor | 1, 13 |

*: temperature-sensitive (CUP when shifted to 25°C)

**: using ssGFP secreted in heat-shock treatment

***: defective in cell lineage of coelomocyte

***: using ANF (rat Atrial Natriuretic Factor)-GFP

n. d: not determined.

**References**

1. Fares, H., and Greenwald, I. (2001). Genetic analysis of endocytosis in *Caenorhabditis elegans*: coelomocyte uptake defective mutants. Genetics 159, 133-145.

2. Schaheen, B., Dang, H., and Fares, H. (2009). Derlin-dependent accumulation of integral membrane proteins at cell surfaces. J. Cell Sci. 122, 2228-2239. doi: 10.1242/jcs.048892

3. Patton, A., Knuth, S., Schaheen, B., Dang, H., Greenwald, I., and Fares, H. (2005). Endocytosis function of a ligand-gated ion channel homolog in *Caenorhabditis elegans*. Curr. Biol. 15, 1045-1050. doi: 10.1016/j.cub.2005.04.057

4. Dang, H., Li, Z., Skolnik, E. Y., and Fares, H. (2004). Disease-related myotubularins function in endocytic traffic in *Caenorhabditis elegans*. Mol. Biol. Cell 15, 189-196. doi:  10.1091/mbc.E03-08-0605

5. Bednarek, E. M., Schaheen, L., Gaubatz, J., Jorgensen, E. M., and Fares, H. (2007). The plasma membrane calcium ATPase MCA-3 is required for clathrin-mediated endocytosis in scavenger cells of *Caenorhabditis elegans*. Traffic 8, 543-553. doi: 10.1111/j.1600-0854.2007.00547.x

6. Xiao, H., Chen, D., Fang, Z., Xu, J., Sun, X., Song, S., Liu, J., and Yang, C. (2009). Lysosome biogenesis mediated by vps-18 affects apoptotic cell degradation in *Caenorhabditis elegans*. Mol. Biol. Cell 20, 21-32. doi: 10.1091/mbc.E08-04-0441

7. Amin, N. M., Shi, H., and Liu, J. (2010). The FoxF/FoxC factor LET-381 directly regulates both cell fate specification and cell differentiation in *C. elegans* mesoderm development. Development 137, 1451-1460. doi: 10.1242/dev.048496

8. Gengyo-Ando, K., Kuroyanagi, H., Kobayashi, T., Murate, M., Fujimoto, K., Okabe, S., and Mitani, S. (2007). The SM protein VPS-45 is required for RAB-5-dependent endocytic transport in *Caenorhabditis elegans*. EMBO Rep. 8, 152-157. doi: 10.1038/sj.embor.7400882

9. Speese, S., Petrie, M., Schuske, K., Ailion, M., Ann, K., Iwasaki, K., Jorgensen, E. M., and Martin, T. F. (2007). UNC-31 (CAPS) is required for dense-core vesicle but not synaptic vesicle exocytosis in *Caenorhabditis elegans*. J. Neurosci. 27, 6150-6162. doi: 10.1523/JNEUROSCI.1466-07.2007

10. Grant, B., Zhang, Y., Paupard, M. C., Lin, S. X., Hall, D. H., and Hirsh, D. (2001). Evidence that RME-1, a conserved *C. elegans* EH-domain protein, functions in endocytic recycling. Nat. Cell Biol. 3, 573-579. doi:10.1038/35078549

11. Sato, M., Sato, K., Liou, W., Pant, S., Harada, A., and Grant, B. D. (2008). Regulation of endocytic recycling by *C. elegans* Rab35 and its regulator RME-4, a coated-pit protein. EMBO J. 27, 1183-1196. doi: 10.1038/emboj.2008.54

12. Sato, M., Sato, K., Fonarev, P., Huang, C. J., Liou, W., and Grant, B. D. (2005). *Caenorhabditis elegans* RME-6 is a novel regulator of RAB-5 at the clathrin-coated pit. Nat. Cell Biol. 7, 559-569.

13. Zhang, Y., Grant, B., and Hirsh, D. (2001). RME-8, a conserved J-domain protein, is required for endocytosis in *Caenorhabditis elegans*. Mol. Biol. Cell 12, 2011-2021. doi: 10.1091/mbc.12.7.2011
